# Dehydroleucodine exerts an antiproliferative effect on human Burkitt’s lymphoma Daudi cells via SLC7A11-mediated ferroptosis

**DOI:** 10.3389/fphar.2025.1572364

**Published:** 2025-04-14

**Authors:** Rui Shen, Fang Cheng, Rui Guo, Wenjing Wang, Xiaolong Yang, Yemiao Chen, Yaokai Chen

**Affiliations:** ^1^ Department of Infectious Diseases, Chongqing Public Health Medical Center, Chongqing, China; ^2^ Biobank, Chongqing Public Health Medical Center, Chongqing, China; ^3^ School of Pharmaceutical Sciences, South-Central Minzu University, Wuhan, China; ^4^ Anhui Province Key Laboratory of Bioactive Natural Products, School of Pharmacy, Anhui University of Chinese Medicine, Hefei, China

**Keywords:** dehydroleucodine, burkitt’s lymphoma, cell death, ferroptosis, solute carrier family 7 member 11

## Abstract

**Background:**

Burkitt’s lymphoma (BL) is a rare, highly aggressive B-cell non-Hodgkin’s lymphoma known for rapid proliferation. While most patients respond well to intensive chemotherapy, those who are older, have comorbidities, or develop therapy resistance show limited outcomes.

**Purpose:**

This study aims to evaluate the *in vitro* anti-tumor activity of dehydroleucodine (DhL), a novel plant-derived chemotherapeutic agent, against BL cells and to elucidate the molecular mechanisms underlying its effects.

**Methods:**

A screening of 42 plant-derived small molecules identified DhL as a potent inhibitor of BL growth. We evaluated DhL’s effects on cell cycle progression, apoptosis, and ferroptosis pathways using cell viability assays, flow cytometry, transcriptomic analysis, and validation experiments.

**Results:**

DhL demonstrated robust and specific anti-proliferative effects against BL Daudi cells. Mechanistic investigations revealed that DhL exerts its effects through cell cycle modulation, induction of apoptosis, and ferroptosis. Transcriptomic analysis identified SLC7A11 as a critical regulator of DhL-mediated ferroptosis, which was further validated experimentally.

**Conclusion:**

DhL shows strong potential as a novel chemotherapeutic agent for BL treatment by targeting SLC7A11-mediated ferroptosis. Further investigation is warranted to confirm its efficacy and clinical utility in diverse BL patient populations.

## 1 Introduction

Burkitt’s lymphoma (BL) is an exceptionally aggressive subtype of B-cell non-Hodgkin’s lymphoma that may manifest in both pediatric and adult populations. The standard treatment regimen for BL is multiagent chemoimmunotherapy, which has achieved cure rates exceeding 90% in children and adolescents (Minard-Colin et al., 2020). However, adults are more susceptible to the toxic effects of dose-intensive chemotherapy treatment. Prospective clinical trials have demonstrated that only 75%–85% of adult patients achieve long-term remission (Dunleavy et al., 2013; Roschewski et al., 2020; Evens et al., 2021), highlighting a significant therapeutic challenge. Furthermore, treatment-related mortality (TRM) and risk of relapse remain alarmingly high among specific patient subsets, including those with therapy-resistant or recurrent disease, older individuals, and those with multiple comorbidities (Crombie and LaCasce, 2021). One recent comprehensive study indicated that intensive chemotherapy may result in TRM rates as high as 10%, with nearly all cases of recurrent or refractory disease proving fatal (Irimajiri et al., 1996). Thus, safer therapeutic agents and novel chemotherapeutic alternatives are required for BL.

Natural products (NPs) are secondary metabolites with molecular weights less than 3,000 Da that are produced by organisms in order to facilitate adaptation to their environment (van Santen et al., 2019), and are known to be a treasure trove for the discovery of novel anti-tumor drugs (Lou et al., 2019; Li et al., 2023). More than 50% of the anti-tumor drugs currently in clinical use are derived from NPs and their derivatives (Das and Agarwal, 2024), and include the widely used anti-tumor drugs resveratrol, paclitaxel, and vincristine. dehydroleucodine (DhL), a sesquiterpene lactone (SL) extracted from the medicinal plant *Artemisia argyi*, has demonstrated anti-inflammatory, antiparasitic, and antimicrobial activities (Gomez et al., 2024). Natalia et al., observed that DhL may induce cell cycle arrest, apoptosis, and DNA damage in human astrocytoma cells by modulation of the p73/p53 signaling pathway (Bailon-Moscoso et al., 2015). Valeria et al., showed that DhL inhibits tumor growth in a murine melanoma model by inducing cell cycle arrest, senescence, and apoptosis (Costantino et al., 2016). Furthermore, Paola et al., showed that DhL may inhibit the proliferation of various acute myeloid leukemia cells (such as HL-60, Kasumi-1, and KG-1), with EC_50_ values within 20 µM (Ordóñez et al., 2020). Thus, much attention has been focused on the potential value of DhL for therapeutic use in different categories of neoplastic disease. However, the effects of DhL on BL cells have not been reported in the literature. Thus, whether DhL has the capacity to inhibit BL cells, and the potential mechanisms whereby DhL may inhibit BL cell growth warrants further investigation.

Ferroptosis, a category of regulated cell death driven by iron-dependent lipid peroxidation (LPO), results from the depletion of intracellular reduced glutathione (GSH) following cysteine deprivation. This depletion renders cells vulnerable to the fenton reaction, where free iron catalyzes the generation of toxic lipid alkoxyl radicals, disrupting redox homeostasis and culminating in oxidative damage to cellular membranes, including those of the cytoplasm and endocytic organelles (Bilgrami et al., 1981; Burmahl, 1999). Accumulating evidence implicates ferroptosis in various facets of tumorigenesis, including initiation, progression, metastasis, and the development of treatment resistance across a range of cancers (Friedmann Angeli et al., 2019; Ubellacker et al., 2020), notably including BL (Wang et al., 2019; Freitas et al., 2024). Artesunate, a semi-synthetic derivative of the sesquiterpene lactone, artemisinin, which is extracted from *Artemisia annua*, has demonstrated anti-proliferative effects against BL cell lines (Daudi and CA-46) by induction of ferroptosis *via* an ATF4-CHOP-CHAC1 dependent pathway (Wang et al., 2019). Furthermore, the acquisition of ferroptosis resistance, as observed in BL xenografts with the accumulation of 7-dehydrocholesterol, has been linked to a more aggressive phenotype (Freitas et al., 2024). These findings underscore the potential for the targeting of ferroptotic pathways as a therapeutic strategy for BL.

In this study, we initially screened a small molecule compound library, and identified a natural product (DhL) as exhibiting cytotoxic activity against various tumor cell lines, with BL Daudi cells demonstrating the highest sensitivity. We subsequently verified the ability of DhL to regulate the cell cycle and induce apoptosis in Daudi cells. Importantly, through transcriptomic analysis, we observed that ferroptosis represents a novel mechanism by which DhL mediates Daudi cell death. Further investigation revealed that the regulation of the ferroptosis by DhL is solute carrier family 7 member 11 (SLC7A11)-mediated. The present data suggest that DhL may potentially be utilized as a novel therapeutic agent for the clinical management of BL.

## 2 Materials and methods

### 2.1 Cell lines and culture

Daudi (human BL cells), SUDHL-2 (diffuse large B-cell lymphoma cells), and SUDHL-4 (B-cell lymphoma cells) cells were purchased from Saibekang Biotechnology (Shanghai, China) and Shenzhen Haodi Huatuo Biotechnology (Shenzhen, China), respectively. A549 (adenocarcinomic human alveolar basal epithelial cells, lung cancer related) cells were gifted by the Southwest Hospital Pathology Key Laboratory (Chongqing, China). Daudi, SUDHL-2, and SUDHL-4 cells were cultured in RPMI 1640 medium (Gibco, MD, United States) supplemented with 10% FBS (Gibco, MD, United States) and 100 U/mL penicillin/streptomycin (Beyotime, Shanghai, China). A549 cells were cultured in DMEM medium (Gibco, MD, United States) supplemented with 10% FBS. All cells were cultured at 37°C in a humidified incubator with 5% CO_2_. Dehydroleucodine (DhL; purity ≥98%, HPLC) was purchased from AbMole (Houston, TX, United States). DhL was dissolved in DMSO (Beyotime, Shanghai, China) to a concentration of 10 mM to create the stock solution, which was then stored at −80°C. Working solutions of DhL were prepared by diluting the stock solution with RPMI 1640 or DMEM medium.

### 2.2 Cell viability assays

Daudi cells (5 × 10^5^ cells/mL) were seeded in 96-well plates with a volume of 90 μL/well. The cells were then treated with DhL (10 μL/well) at various concentrations (0, 5, 10, 15, 20, 25, and 30 μM) for 24 and 48 h. SUDHL-2 and SUDHL-4 cells (5 × 10^5^ cells/mL), and A549 cells (3 × 10^4^ cells/mL) were treated with 30 μM DhL for 48 h. Subsequent to treatment, 10 μL of CCK-8 reagent (Beyotime, Shanghai, China) was added to each well, and the cells were incubated at 37°C for 1 h. Absorbance was then measured at 450 nm using a spectrophotometer.

### 2.3 Cell cycle assays

Cells (5 × 10^5^ cells/well) were plated in 12-well plates and treated with the indicated concentrations of DhL. After 24 h, cells were harvested, pre-treated, and resuspended in 70% pre-cooled ethanol in PBS overnight at 4°C. Subsequently, the cells were centrifuged at 1,000 *g*, washed with 1 mL pre-cooled PBS, resuspended, and stained with 500 μL propidium iodide staining solution (Beyotime, Shanghai, China) according to the manufacturer’s instructions. Cell cycle analysis was performed using flow cytometry (BD Accuri^®^ C6, USA).

### 2.4 Annexin V-FITC/PI apoptosis assay

Apoptosis was measured using the Annexin V-FITC/PI double-staining kit (Beyotime, Shanghai, China). Briefly, Daudi cells were cultured in 12-well plates and treated with 5, 10, and 15 µM DhL for 48 h at 37°C. Subsequent to treatment, cells were collected, washed with PBS, and resuspended. Aliquots of 5 × 10^4^ to 1 × 10^5^ cells were then resuspended in 195 μL of Annexin V-FITC binding solution. Subsequently, 5 µL of Annexin V-FITC and 10 µL of PI were added, and the cells were incubated for 20 min in the dark at room temperature, following the manufacturer’s instructions. After filtration, apoptotic cells were detected using flow cytometry (BD Accuri^®^ C6, USA).

### 2.5 Measurement of mitochondrial membrane potential (ΔΨm)

Mitochondrial membrane potential (MMP) levels were assessed using the JC-1 dye (Beyotime, Shanghai, China). Daudi cells were seeded in 12-well plates and treated with various concentrations of DhL for 24 h. After treatment, cells were harvested, washed twice with PBS, and incubated with JC-1 working solution for 20 min at 37°C in a cell incubator. Fluorescence intensity was measured using flow cytometry (BD Accuri^®^ C6, USA). FlowJo software (BD Accuri^®^ C6, USA) was employed for further data analysis.

### 2.6 Transmission electron microscopy (TEM) assays

Daudi cells were treated with 10 μM DhL for 48 h. Cells were then harvested, washed with PBS, and centrifuged at 1,000 rpm for 5 min. Cells were fixed with glutaraldehyde solution (Solarbio, Shanghai, China) overnight.

### 2.7 Reactive oxygen species (ROS) test by H2DCFDA

Cells (5 × 10^5^ cells/well) were treated with 5 and 10 μM DhL for 2 h. For ferroptosis inhibitor pretreatment, cells were pretreated with the inhibitors for 2 h before DhL exposure. Ferrostatin-1 (0.1,1,5 μM) was then added to all groups, and cells were incubated for 48 h. Cells were stained with 10 μM DCFH-DA (Beyotime, Shanghai, China) in RPMI 1640.

### 2.8 Detection of LPO levels

Cells were treated with DhL or vehicle (DMSO) for 48 h and then incubated with 5 μM C11-BODIPY 581/591 (Invitrogen, Carlsbad, CA, United States) in serum-free RPMI-1640 medium for 30 min at 37°C in the dark. Cells were then washed thoroughly with PBS (at least twice). Lipid ROS levels were analyzed using flow cytometry.

### 2.9 Measurement of GSH

Total GSH levels in cell lysates were measured using a GSH detection kit (Beyotime, Shanghai, China) according to the manufacturer’s instructions.

### 2.10 Measurement of malondialdehyde (MDA)

Cells were lysed in RIPA Lysis Buffer (Beyotime, Shanghai, China) on ice for 30 min. The lysate was centrifuged at 16,000 g for 15 min. One aliquot of the supernatant was used for protein concentration determination using a BCA assay, while another aliquot was used for MDA measurement using the MDA detection kit (Beyotime, Shanghai, China) according to the manufacturer’s instructions.

### 2.11 RNA-seq (sequencing) and analysis

Daudi cells (4.5 × 10^5^ cells/mL) were seeded in T25 flasks and exposed to 10 μM DhL for 48 h. Total mRNA was extracted using Trizol reagent (Thermo Fisher Scientific, Carlsbad, CA, United States) according to the manufacturer’s instructions and, sent to the Beijing Allwegene Company for whole-transcriptome sequencing and analysis. Briefly, 1.5 μg of RNA per sample was used as input for RNA sample preparation. mRNA was purified from total RNA using poly-T oligo-attached magnetic beads. Raw sequences were processed to obtain clean reads, which were then mapped to the reference genome sequence using STAR software. Differential expression analysis between DhL-treated and control groups was performed using the DESeq R package (version 1.10.1). Genes with an adjusted *p*-value < 0.05 were considered differentially expressed (DEGs). Gene Ontology (GO) enrichment analysis of DEGs was performed using the GOseq R package based on the Wallenius non-central hyper-geometric distribution, which adjusts for gene length bias.

### 2.12 Western blotting assays

Daudi cells were washed with cold PBS and lysed with ice-cold RIPA buffer (Beyotime, Shanghai, China) supplemented with PMSF (Beyotime, Shanghai, China), protease inhibitors (Beyotime, Shanghai, China), and phosphatase inhibitors (Beyotime, Shanghai, China). Protein concentrations in cell lysates were determined using the BCA Assay Kit (Thermo Fisher Scientific, Carlsbad, CA, United States). Equal amounts of protein were separated by SDS-PAGE and transferred onto PVDF membranes (Merck Millipore, Darmstadt, Germany). Membranes were blocked with QuickBlock™ Western Blocking Buffer (Beyotime, Shanghai, China) and probed with specific primary and secondary antibodies. The following primary antibodies were used, *viz.*, GPX4 (ab125066, 1:1,000 dilution, Santa Cruz Biotechnology, SLC7A11 (ab175186, 1:1,000 dilution, Santa Cruz Biotechnology), FTH1 (sc-376594, 1:1,000 dilution, Santa Cruz Biotechnology), and ACTB/β-actin (AA128, 1:500 dilution, Beyotime).

### 2.13 Molecular docking simulation

The SDF file of the primary active component for the core drug was obtained from the PubChem database, while the structures of the key target proteins were sourced from the PDB database. The target was optimized in PyMOL 2.1.0 by removing water molecules and small-molecule ligands. Subsequently, hydrogen atoms and charges were processed using AutoDock Tools 1.5.6, and the results were saved in pdbqt format. With the key target acting as the receptor and the corresponding active components as ligands, molecular docking was performed using Vina 2.0 from the PyRx software package to calculate binding energy and produce output results. Finally, the results were visualized using PyMOL (https://pymol.org/2/). The Affinity (kcal/mol) value indicates the binding strength; lower values signify a more stable ligand-receptor interaction. Additionally, 2D diagrams were created using Discovery Studio 2020 Client (https://discover.3ds.com/discovery-studio-visualizer-download).

### 2.14 Statistical analysis

All graphs were created, and statistical analyses were performed using GraphPad Prism 9.0 (GraphPad Software, Inc.). One-way ANOVA was used for statistical comparisons. A *p-value* < 0.05 was considered statistically significant.

## 3 Results

### 3.1 DhL exhibits a significant inhibitory effect on daudi cells

**FIGURE 1:**
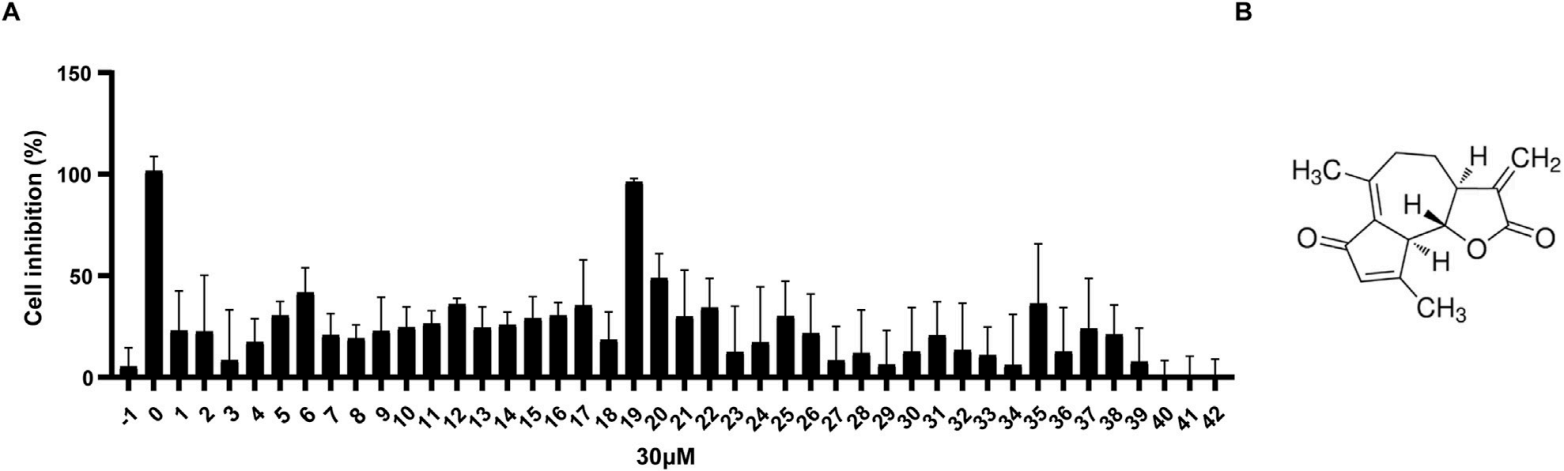
DhL exhibits a significant inhibitory effect on Daudi cells. **(A)** Cell viability of Daudi cells after treatment with 30 µM molecules (YXL-1–42) for 48 h; −1, 0.3%DMSO; 0, 30 µM Adriamycin. **(B)** The chemical structure of YXL-19 (dehydroleucodine, DhL) (C_15_H_16_O_3_).

A library of 42 small molecules (YXL-1 to YXL-42) derived from medicinal plants and their endophytic fungi (established by our laboratory previously), was screened for anti-BL activity against Daudi cells. The impact of 42 small molecules at 30 µM on Daudi cells viability was evaluated using the CCK-8 assay. Compound YXL-19, isolated from *A. argyi*, demonstrated potent inhibitory activity, exceeding 50% inhibition (Figure 1A), and comparable to the positive control, doxorubicin. The chemical structure of YXL-19 is depicted in Figure 1B and corresponds to a molecular formula of C_15_H_16_O_3_. A search in the PubChem database identified this compound as DhL, an SL initially isolated from the medicinal plant *Cirsium japonicum*. This observation identifies DhL as a promising candidate for further anti-tumor investigation.

### 3.2 DhL inhibits proliferation, and induces cell cycle arrest and apoptosis in daudi cells

**FIGURE 2:**
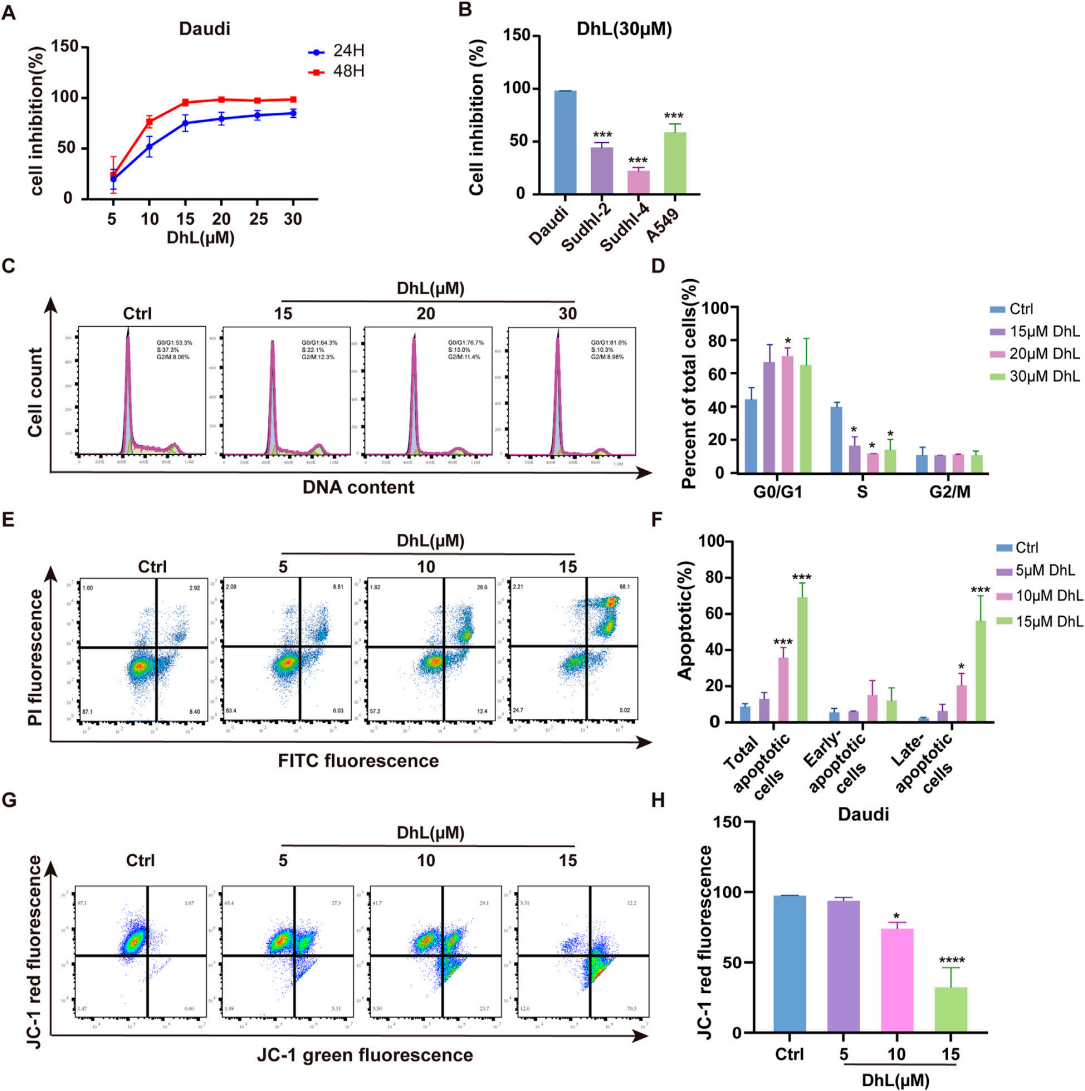
DhL inhibits proliferation, induces cell cycle arrest and apoptosis in Daudi cells. **(A)** Cell inhibition rate of Daudi cells treated with different concentrations of DhL for 24 and 48 h. **(B)** Cell inhibition rate of Daudi, SUDHL-2, SUDHL-4, and A549 cells treated with 30 μM DhL for 48 h **(C, D)** Cell cycle of Daudi cells treated with different concentrations of DhL for 24 h. Image **(C)** and quantitative analysis for apoptotic cells **(D)** are presented. **(E, F)** Levels of apoptosis in Daudi cells treated with different concentrations of DhL for 48 h, flow cytometry **(E)** and quantitative analysis of the apoptotic cells **(F)** utilized the FITC-PI dye. **(G, H)** Trends in mitochondrial membrane potential of Daudi cells treated with different concentrations of DhL for 24 h, subsequent to which the cells stained with JC-1 were detected using flow cytometry **(G)** and quantitative analysis **(H)**. DhL, dehydroleucodine; Ctrl, control; Data from three independent experiments are shown as mean ± SD. ^*^ *p* < 0.05, ^**^ *p* < 0.01, ^***^ *p* < 0.001, ^****^ *p* < 0.0001 vs. Ctrl group.

The anti-proliferative activity of DhL against BL was evaluated in Daudi cells. Cells were treated with increasing concentrations of DhL (0, 5, 10, 15, 20, 25, and 30 μM), and viability was assessed using the CCK-8 assay (Figure 2A). DhL exhibited a dose- and time-dependent reduction in Daudi cell viability, indicating the cytotoxic potential of DhL. The calculated IC_50_ values for DhL at 24h and 48 h were 9.022 μM and 8.596 μM, respectively. Importantly, DhL demonstrated greater anti-proliferative effects in Daudi cells compared to other cancer cell lines (SUDHL-2, SUDHL-4, and A549), suggesting a potential selective proclivity for BL cells (Figure 2B).

To explore the mechanism of DhL-induced growth inhibition, cell cycle analysis was performed. Treatment with DhL resulted in G0/G1 phase arrest in Daudi cells after 24 h, consistent with results from previous reports on HeLa cells (Costantino et al., 2013) (Figures 2C, D). Furthermore, Annexin V-FITC assays revealed a dose-dependent increase in both early and late apoptotic cells following DhL treatment (Figures 2E, F). Flow cytometry analysis of JC-1 staining confirmed a reduction in MMP in DhL-treated cells after 24 h (Figures 2G, H), suggesting the involvement of the mitochondrial-mediated apoptotic pathway. These findings collectively demonstrate that DhL exerts its anti-tumor effects in Daudi cells through the induction of cell cycle arrest and apoptosis, potentially *via* mitochondrial dysfunction.

### 3.3 DhL induces altered gene expression in Daudi cells

**FIGURE 3:**
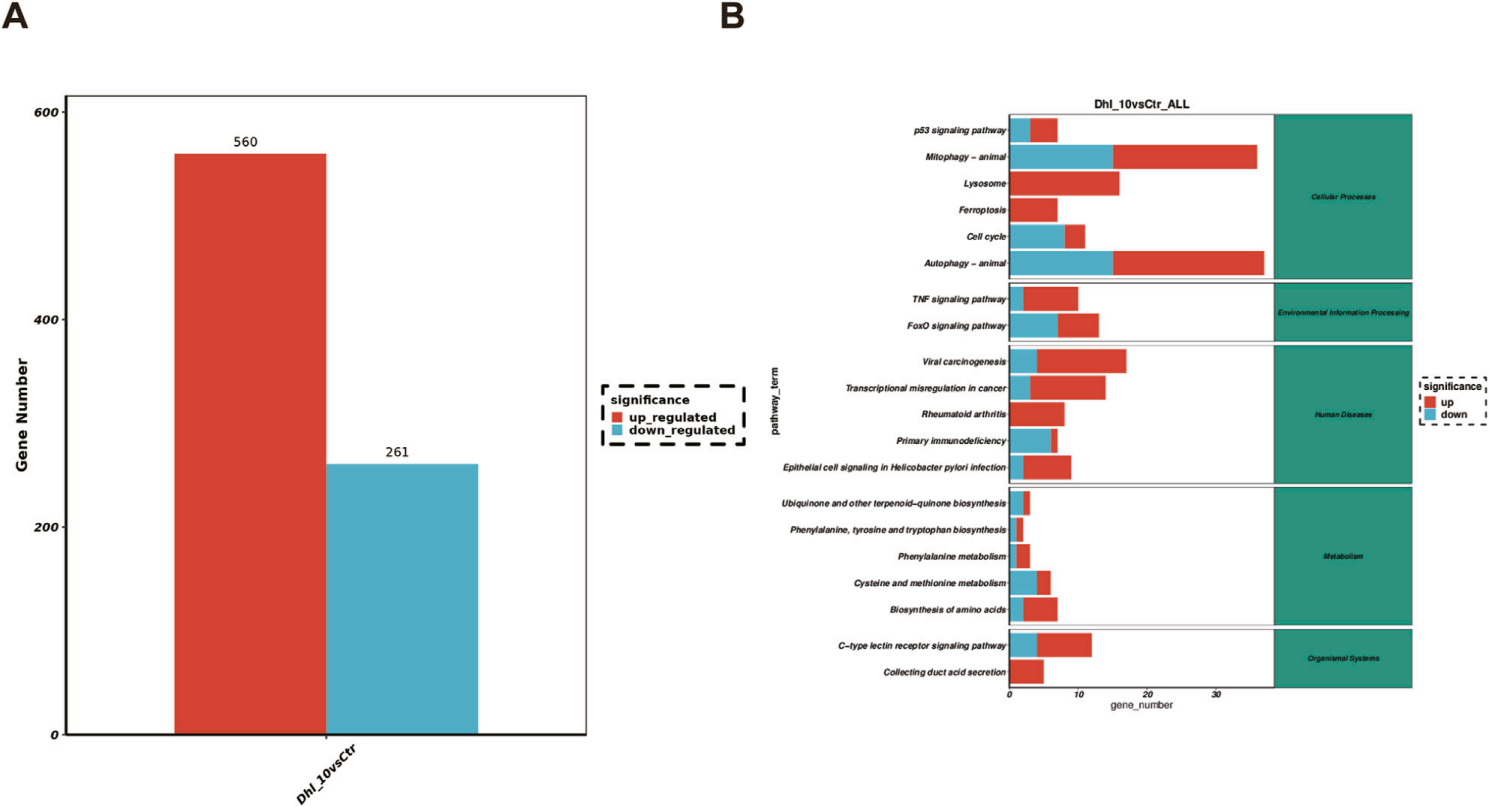
DhL induces altered gene expression in Daudi cells. **(A)** Differences in RNA expression caused by DhL treatment. **(B)** Enrichment analysis of KEGG pathway. DhL, dehydroleucodine; ^*^ *p*˂0.05, ^**^ *p*˂0.01, ^***^ *p*˂0.001 vs. Ctrl group.

To investigate the global transcriptional effects of DhL on Daudi cells, RNA sequencing (RNA-Seq) was performed. Following treatment with 10.0 μM DhL for 48 h, 560 transcripts were upregulated and 261 transcripts were downregulated when compared to the control (Figure 3A). Subsequent pathway enrichment analysis of these DEGs provided insights into the molecular mechanisms underlying DhL activity. Kyoto Encyclopedia of Genes and Genomes (KEGG) pathway analysis identified ferroptosis as one of the significantly enriched pathways modulated by DhL (Figure 3B). The complete RNA-Seq dataset for our study has been deposited in the Sequence Read Archive (SRA) database under accession number PRJNA1201049 (https://www.ncbi.nlm.nih.gov/sra), and is publicly accessible. This data provides a valuable resource for further investigation of the molecular mechanisms underlying DhL-induced ferroptosis in Daudi cells.

### 3.4 DhL induces ferroptosis *in vitro*

**FIGURE 4:**
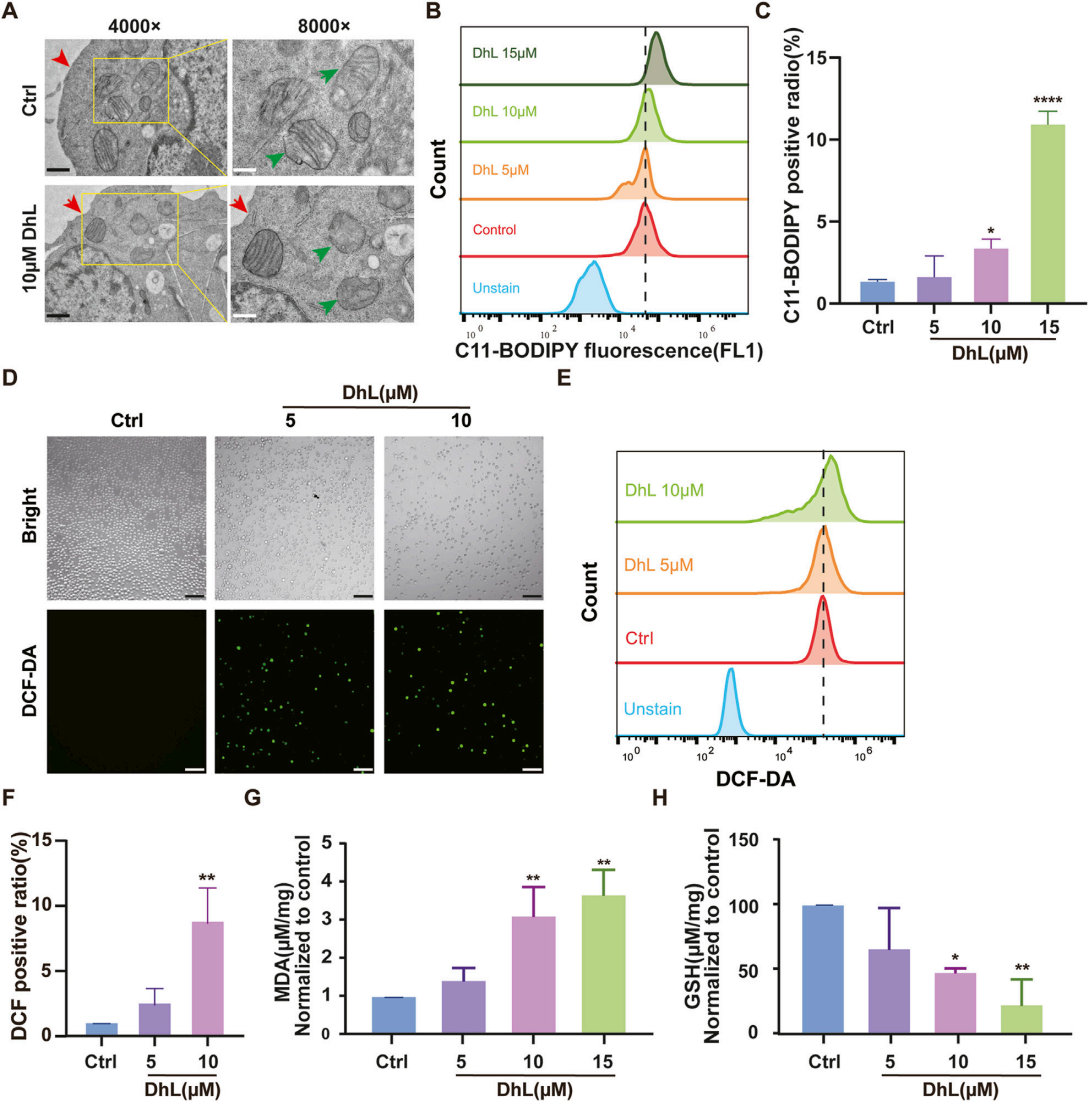
DhL induces ferroptosis *in vitro*. **(A)** Transmission electron micrographs of DhL-induced changes in the structure of Daudi cells. The red arrow indicates damaged membrane and the green indicates the destruction of mitochondrial cristae. Black scale bar indicates 1 μm, white scale bar indicates 500 nm. **(B, C)** Effect of different concentrations of DhL on Daudi cells for 48 h, and subsequently tested by C11-BODIPY lipid probe. Flow cytometry **(B)** and quantitative analysis for lipid peroxidation **(C)** are presented. **(D–F)** DhL (5/10/15 μM) was applied to Daudi cells for 48 h. ROS levels were then detected using DCF probes for fluorescence microscopy **(D)**, flow cytometry **(E)**, and quantitative analysis **(F)**. **(G)** Effect of different concentrations of DhL on MDA levels in Daudi cells for 48 h. **(H)** Effect of different concentrations of DhL on GSH levels in Daudi cells. Data from three independent experiments are shown as mean ± SD; Scale bar indicates 130 μm; DhL, dehydroleucodine; Ctrl, control; ROS, reactive oxygen species; MDA, malondialdehyde; GSH, glutathione; Data from three independent experiments are shown as mean ± SD. ^*^ *p* < 0.05, ^**^ *p* < 0.01, ^***^ *p* < 0.001 vs. Ctrl group.

To further verify whether DhL induces ferroptosis in Daudi cells, we examined several key markers of this process. As illustrated in Figure 4A, cells treated with DhL exhibited characteristic subcellular morphological changes associated with ferroptosis, including the absence of cell membrane vesicles or ruptures, smaller or more shriveled mitochondria, and reduced or missing mitochondrial cristae (Xia et al., 2020). These morphological alterations are consistent with established features of ferroptosis.

The induction of LPO, a hallmark of ferroptosis (Xu et al., 2021), was assessed using the C11-BODIPY probe. Flow cytometry analysis revealed a dose-dependent increase in lipid ROS levels following treatment with 5, 10, and 15 μM DhL (Figures 4B, C). Similarly, total ROS levels were also elevated in a dose-dependent manner, as measured by both flow cytometry and fluorescence microscopy (Figures 4D–F).

Also, MDA levels, which is a marker of LPO(Nazir et al., 2020), were increased (Figure 4G), while GSH levels, which is an important antioxidant (Petrou et al., 2018), were significantly decreased (Figure 4H) following treatment with 5, 10, and 15 μM DhL, further supporting evidence of the induction of ferroptosis. These outcomes are consistent with the KEGG pathway analysis, and provide strong evidence that DhL triggers ferroptosis in Daudi cells.

### 3.5 DhL-induced Daudi cell death is abrogated by inhibition of ferroptosis

To confirm the role of ferroptosis in DhL-induced cell death, we utilized Ferrostatin-1 (Fer-1), a known potent inhibitor of ferroptosis. Fer-1 effectively blocks LPO without affecting mitochondrial ROS production or lysosomal membrane permeability (Zhang et al., 2021), and has been shown to inhibit erastin-induced ferroptosis in HT-1080 cells with an EC_50_ of 60 nM (Laukaitiene et al., 2023).

**FIGURE 5:**
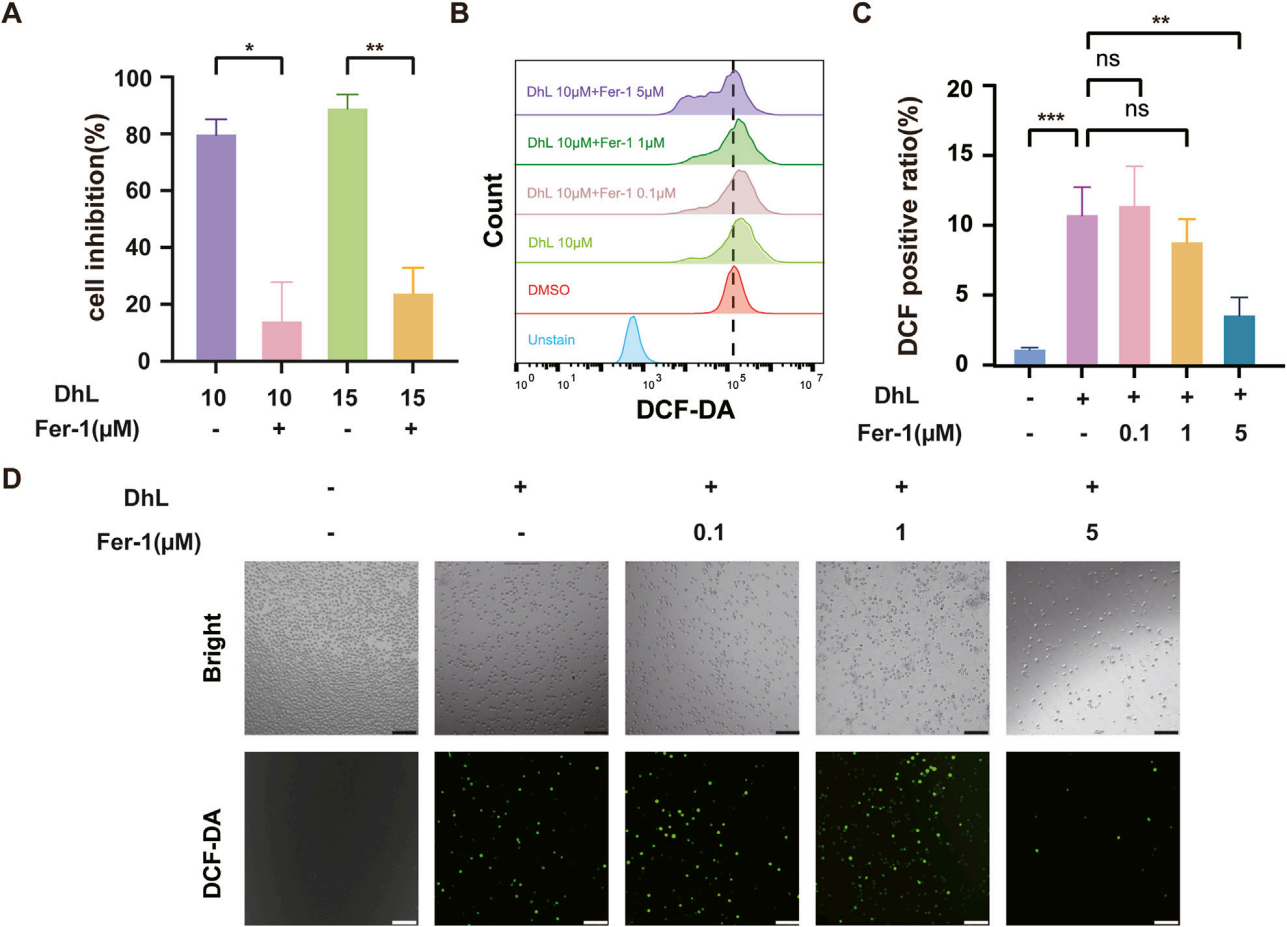
DhL-induced Daudi cell death is abrogated by inhibition of ferroptosis. **(A)** Cell inhibition rate after pretreatment of Daudi cells with 5 μM Fer-1 for 2 h, followed by 48 h of co-incubation with 10 or 15 μM DhL. **(B–D)** The Daudi cells were treated with different concentrations of Fer-1 for 2 h, followed by 10 μM DhL and co-incubated for 48 h. The total ROS levels were detected by flow cytometry **(B)**, quantitative analysis for ROS levels **(C)** and fluorescence microscope **(D)** are presented, respectively. Scale bar indicates 130 μm; DhL, dehydroleucodine; Fer-1, ferrostatin-1; ns, no significant difference; Data from three independent experiments are shown as mean ± SD. ^*^ *p*˂0.05, ^**^ *p*˂0.01, ^***^ *p*˂0.001.

Daudi cells were pre-treated with Fer-1 prior to DhL exposure. Fer-1 was observed to significantly reduce DhL-induced cell death, as evidenced by increased cell viability (Figure 5A). Furthermore, Fer-1 treatment was observed to reduce DhL-induced ROS accumulation, as measured by flow cytometry (Figures 5B, C) and fluorescence microscopy (Figure 5D). These results demonstrate that Fer-1 mitigates the effects of DhL by inhibition of ferroptosis, confirming the involvement of the ferroptotic cell death pathway in the anti-tumor activity shown by DhL.

### 3.6 Daudi cell death is synergistically induced by DhL and HG106

**FIGURE 6:**
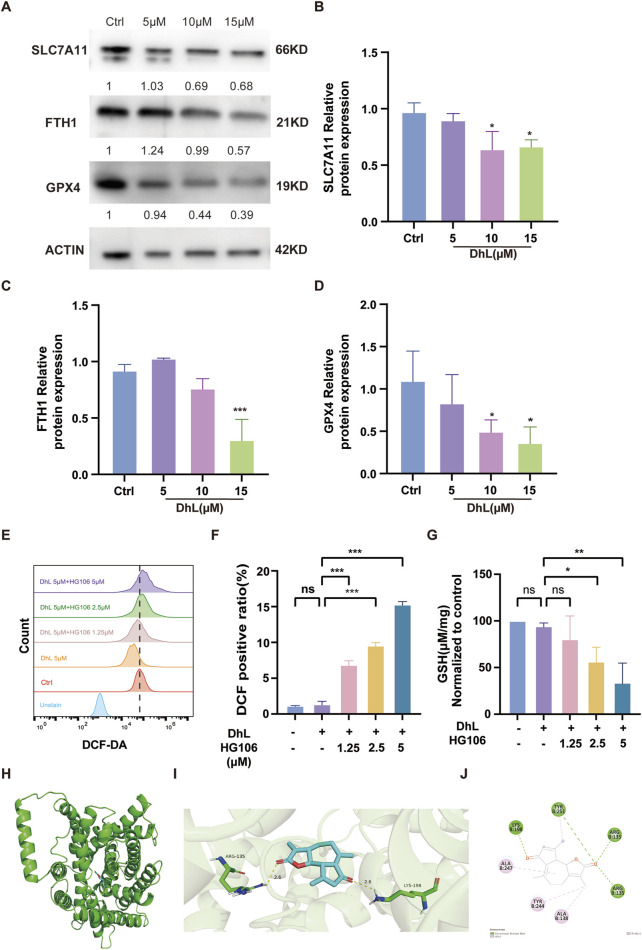
Daudi cell death is synergistically induced by DhL and HG106. **(A–D)** The changes in ferroptosis-related proteins (FTH1, SLC7A11, GPX4) were tested by Western blot after treatment of Daudi cells with 5, 10, and 15 μM DhL for 48 h. Western blot bands of proteins **(A)**. The internal control is actin. The quantitative analysis for protein expression of SLC7A11 **(B)**, FTH1 **(C)** and GPX4 **(D)** are presented, respectively. **(E, F)** Daudi cells were treated with 5 μM DhL and Fer-1 for 48 h, flow cytometry **(E)** and quantitative analysis **(F)** for ROS levels are presented, respectively. **(G)** GSH levels in Daudi cells after 48 h of co-administration of 5 μM DhL and different concentrations of HG106 (the SLC7A11 inhibitor). The overall view **(H)**, pocket view **(I)**, and 2D diagram **(J)** of DhL binding to SLC7A11 are presented, respectively. DhL, dehydroleucodine; Ctrl, control; FTH1, ferritin heavy chain 1; SLC7A11, solute carrier family 7 member 11; GPX4, glutathione peroxidase 4; GSH, glutathione; ns, no significant difference; Data from three independent experiments are shown as mean ± SD. ^*^ *p* < 0.05, ^**^ *p* < 0.01, ^***^ *p* < 0.001 vs. Ctrl group.

DhL treatment was observed to result in decreased expression of ferroptosis-related proteins (Figures 6A–D), including ferritin heavy chain 1 (FTH1), SLC7A11, and glutathione peroxidase 4 (GPX4). To further investigate the role of SLC7A11, we used the SLC7A11 inhibitor, HG106, which blocks cystine uptake and reduces intracellular GSH synthesis (Hu et al., 2020). Combined treatment with DhL and HG106 resulted in a dose-dependent increase in total ROS levels (Figures 6E, F) and a significant decrease in GSH levels (Figure 6G) in Daudi cells. These observations suggested a synergistic effect between DhL and HG106 in induction of ferroptosis and inhibition of Daudi cell proliferation. The observed decrease in GSH and increase in ROS further highlights the interplay between DhL, SLC7A11, and ferroptosis in mediating Daudi cell death. What’s more, we performed molecular docking simulation analysis on DhL and SLC7A11. The calculated docking energy of −8 kcal/mol suggests a strong binding affinity between the two molecules. The overall view (Figure 6H), pocket view (Figure 6I), and 2D diagram (Figure 6J) of DhL binding to SLC7A11 are presented. In summary, the results suggest that DhL may directly interact with SLC7A11 to induce ferroptosis, thus inhibiting the proliferation of Daudi cells.

## 4 Discussion

Current treatment modalities for BL remain suboptimal in elderly patients, in individuals with comorbidities, and in those exhibiting treatment intolerance, underscoring the need for both a deeper understanding of the molecular mechanisms which underlie chemotherapeutic efficacy, and the development of novel alternative chemotherapeutic drugs. Our work illustrates previously unrecognized anti-BL activity of the small molecule compound called DhL, and provides initial insights into its mechanism of action, which we have observed to involve modulation of cell cycle progression, apoptosis, and notably, ferroptosis.

The small molecule DhL, identified in the present study, belongs to the SL molecular class, characterized by the α-methylene-γ-lactone moiety, a key structural feature contributing to the antitumor activity of SLs. SLs, a class of terpenoids with diverse biological activities, including anti-inflammatory, antitumor, and antioxidant properties (Perri et al., 2019; Laurella et al., 2022), are widely distributed in the *Asteraceae* and other plant families (Rolnik and Olas, 2021). DhL has previously demonstrated efficacy in gastric ulcer models and notably inhibits preadipocyte differentiation through cell cycle arrest (Abood et al., 2018; Vera et al., 2021). Furthermore, DhL exhibits superior antitumor activity compared to amino derivatives in acute myeloid leukemia cell lines (Ordóñez et al., 2020) In addition to the core SL pharmacophore, DhL possesses an α, β-unsaturated hydroxyl group with an adjacent methyl substituent, a structural element known to influence the potency of small molecule compounds (Feng et al., 2020). Based on these prior observations and our drug screening results, we investigated the specific mechanism whereby DhL inhibits the growth of BL cells.

Our observations reveal that DhL exhibits significantly lower inhibitory activity against non-BL cell lines, such as the A549, SUDHL-2, and SUDHL-4 cell lines (Figure 2B), while demonstrating pronounced antiproliferative activity against BL cell lines, suggesting a degree of selectivity for BL cells. Apoptosis is a primary mechanism by which anticancer drugs inhibit tumor growth (Newton et al., 2019; Wang et al., 2020; 2024), and tumor cell cycle arrest is a key indicator of antitumor efficacy (Wang et al., 2012; Fu et al., 2022). In the present study, DhL induced a concentration-dependent increase in the overall apoptosis rate, with a particularly marked increase in the proportion of cells undergoing late apoptosis. Interestingly, DhL significantly decreased MMP, suggesting that DhL-induced apoptosis in BL cells may be mediated, at least in part, by mitochondrial dysfunction. Transcriptome sequencing analysis, using GO and KEGG pathway enrichment, indicated that the antiproliferative effects of DhL in BL cells involve not only apoptosis induction through MMP disruption but also the potential induction of ferroptosis. Consequently, we further investigated and verified the induction of ferroptosis by DhL in BL cells, and explored the potential underlying mechanisms that are likely to be fundamental to this process.

Ferroptosis, a recently recognized category of regulated cell death, is implicated in the pathogenesis of various diseases (Gao et al., 2021; Long et al., 2024). Studies have shown that Fer-1 effectively inhibits ferroptosis, surpassing the efficacy of phenolic antioxidants to arrest ferroptosis (Miotto et al., 2020). Here, we confirm that DhL induces ferroptosis in BL cells by demonstrating the ability of Fer-1 to mitigate DhL-mediated antiproliferative activity and attenuate DhL-induced ROS accumulation. DhL treatment results in a dose-dependent increase in intracellular ROS, MDA, and LPO, coupled with a decrease in GSH levels. These biochemical changes are accompanied by the characteristic morphological features of ferroptosis, including mitochondrial shrinkage, increased membrane density, and reduction or disappearance of cristae. These data suggest that oxidative stress may be a common pathway underlying both DhL-induced apoptosis and ferroptosis in BL cells. The accumulation of intracellular ferrous iron (Fe^2+^) is another hallmark of ferroptosis (Fu et al., 2023; Chen et al., 2024). Under normal conditions, Fe^2+^ is sequestered by FTH1 to maintain iron homeostasis (Mi et al., 2023). However, iron overload may impair FTH1 function (Wang et al., 2017; Aalikhani et al., 2021). The observed downregulation of FTH1 expression by DhL further supports the induction of ferroptosis by DhL in BL cells. Importantly, we demonstrated synergistic enhancement of apoptosis and ferroptosis in BL cells upon co-treatment with DhL and an SLC7A11 inhibitor (HG106), suggesting a potential therapeutic strategy for BL.

SLC7A11, a critical component of the cystine/glutamate antiporter system Xc-, plays an increasingly recognized role in neoplastic disease (Li et al., 2024). In BL, SLC7A11 expression and function may significantly influence tumor cell survival, proliferation, and therapeutic resistance. Our results demonstrate that DhL inhibits SLC7A11 expression in BL cells, thereby impairing cystine uptake and its subsequent intracellular reduction to cysteine, a precursor for GSH synthesis (Wang et al., 2022; 2023). As a major intracellular antioxidant, GSH is essential for scavenging lipid peroxides and maintaining redox homeostasis (Yang et al., 2022; Wu et al., 2023). Consequently, SLC7A11 inhibition by DhL disrupts the antioxidant defense mechanisms of BL cells, rendering them vulnerable to oxidative stress-induced cell death, including ferroptosis. These observations highlight SLC7A11 as a potential therapeutic target in patients with BL. Targeting SLC7A11, either through direct inhibition of its transporter activity or by depletion of its substrates, may induce ferroptosis in BL cells. This therapeutic strategy may not only directly eliminate tumor cells but also enhance antitumor immunity and modulate the tumor microenvironment. Further investigation into the precise role of SLC7A11 in BL and the development of targeted therapeutic strategies against SLC7A11 are warranted in the future in order to advance the treatment of this aggressive malignancy.

Despite our promising observations, several limitations of the present study must be acknowledged. First, our investigation was primarily confined to the examination of the ferroptosis-inducing potential of DhL in Daudi cells under *in vitro* conditions. Further comprehensive studies are warranted to elucidate the detailed underlying molecular mechanisms and validate the therapeutic efficacy of DhL in animal models. Additionally, although we demonstrated the concurrent activation of both apoptotic and ferroptotic pathways following DhL treatment, the intricate interplay and potential synergistic relationship between these 2 cell death modalities remains to be fully elucidated. Future investigations focusing on the preceding aspects would provide valuable insights into the therapeutic applications that may be possible for DhL.

In conclusion, the present study identified a novel natural small molecule compound, i.e., DhL, which has potent antitumor activity against BL cells, and observed ferroptosis as a novel mechanism underlying the cytotoxic effects of DhL. The ability of DhL to leverage elevated ROS levels in cancer cells, coupled with the potential of DhL for targeted therapy against cancers that overexpress antioxidant molecules such as SLC7A11, positions DhL as a promising candidate for further preclinical development. These observations contribute significantly to the ongoing exploration of the induction of ferroptosis as a novel therapeutic strategy in patients with cancer.

## Data Availability

The datasets presented in this study can be found in online repositories. The names of the repository/repositories and accession number(s) can be found in the article/supplementary material.

## Abbreviations

BL, Burkitt’s lymphoma; DhL, dehydroleucodine; TRM, treatment-related mortality; LPO, lipid peroxidation; GSH, glutathione; SL, sesquiterpene lactone; SLC7A11, solute carrier family 7 member 11; MMP, mitochondrial membrane potential; TEM, transmission electron microscopy; ROS, reactive oxygen species; MDA, malondialdehyde; DEGs, differentially expressed genes; GO, Gene Ontology; RNA-Seq, RNA sequencing; KEGG, Kyoto Encyclopedia of Genes and Genomes; SRA, Sequence Read Archive; Fer-1, Ferrostatin-1; FTH1, ferritin heavy chain 1; GPX4, glutathione peroxidase 4; SHK, Shikonin.
